# Evolution of Multilevel Social Systems in Nonhuman Primates and Humans

**DOI:** 10.1007/s10764-012-9618-z

**Published:** 2012-07-18

**Authors:** Cyril C. Grueter, Bernard Chapais, Dietmar Zinner

**Affiliations:** 1 School of Anatomy, Physiology and Human Biology, The University of Western Australia, Crawley, WA 6009 Australia; 2 Anthropological Institute and Museum, University of Zürich-Irchel, CH-8057 Zürich, Switzerland; 3 Department of Anthropology, University of Montreal, Montréal, Quebec H3C 3 J7 Canada; 4 Cognitive Ethology Laboratory, German Primate Center (DPZ), 37077 Göttingen, Germany; 5 Courant Research Centre “Evolution of Social Behavior”, University of Göttingen, 37077 Göttingen, Germany

**Keywords:** Colobines, Hominins, Modular society, Papionins, Phylogeny, Socioecology

## Abstract

Multilevel (or modular) societies are a distinct type of primate social system whose key features are single-male–multifemale, core units nested within larger social bands. They are not equivalent to fission–fusion societies, with the latter referring to routine variability in associations, either on an individual or subunit level. The purpose of this review is to characterize and operationalize multilevel societies and to outline their putative evolutionary origins. Multilevel societies are prevalent in three primate clades: papionins, Asian colobines, and hominins. For each clade, we portray the most parsimonious phylogenetic pathway leading to a modular system and then review and discuss likely socioecological conditions promoting the establishment and maintenance of these societies. The multilevel system in colobines (most notably *Rhinopithecus* and *Nasalis*) has likely evolved as single-male harem systems coalesced, whereas the multilevel system of papionins (*Papio hamadryas*, *Theropithecus gelada*) and hominins most likely arose as multimale–multifemale groups split into smaller units. We hypothesize that, although ecological conditions acted as preconditions for the origin of multilevel systems in all three clades, a potentially important catalyst was intraspecific social threat, predominantly bachelor threat in colobines and female coercion/infanticide in papionins and humans. We emphasize that female transfers within bands or genetic relationships among leader males help to maintain modular societies by facilitating interunit tolerance. We still lack a good or even basic understanding of many facets of multilevel sociality. Key remaining questions are how the genetic structure of a multilevel society matches the observed social effort of its members, to what degree cooperation of males of different units is manifest and contributes to band cohesion, and how group coordination, communication, and decision making are achieved. Affiliative and cooperative interunit relations are a hallmark of human societies, and studying the precursors of intergroup pacification in other multilevel primates may provide insights into the evolution of human uniqueness.

## Introduction

Among primates, multilevel social systems comprise several hierarchical tiers that are perhaps better referred to as modular or nested systems. The basal unit is typically a spatiotemporally cohesive one-male unit (OMU) with one adult male and one to several females. These core units aggregate at varying temporal scales and in that way form at least one second (the band) or even higher grouping level (the troop or herd) (Grueter and Zinner 2004). Interactions among individuals occur both within and between the social layers, but relationships are clearly much more close knit within the first tier, and core units also tend to represent the reproductive units (Colmenares 2004; Dunbar and Dunbar 1975; Grueter *et al*. in press-b; Stammbach 1987; Yeager and Kirkpatrick 1998; Zhang *et al*. 2012). Several solitary males may also be members of a band or form all-male units (AMUs) that are often loosely attached to the bands (Dunbar and Dunbar 1975; Ren *et al*. 2000; Swedell 2011).

The primate species whose social systems have been described as multilevel include several colobine, most notably snub-nosed monkeys (*Rhinopithecus* spp.), proboscis monkeys (*Nasalis larvatus*), and the papionin species hamadryas baboons (*Papio hamadryas*) and geladas (*Theropithecus gelada*) as well as humans. Circumstantial evidence indicates that multilevel systems may occur in a few more taxa such as mandrills (*Mandrillus sphinx*) and uakaris (*Cacajao* spp.) (Table I). However, a more definitive conclusion on the type of sociality of these latter species will have to await the collection of more fine-grained observational data.

**Table I:** The taxonomic distribution of multilevel socialities among primates

Taxon	References
*Primate species for which multilevel social organization has been described*	
Snub-nosed monkeys (*Rhinopithecus bieti*, *R*. *roxellana*, *R*. *brelichi*, *R*. *avunculus*)	Kirkpatrick 1998; Kirkpatrick and Grueter 2010; Zhang *et al*. 2006
Proboscis monkey (*Nasalis larvatus*	Yeager 1990; see also Matsuda *et al*. 2010
Black-shanked douc langur (*Pygathrix nigripes*)	Hoang 2007; Rawson 2009
Gelada baboon (*Theropithecus gelada*)	Dunbar and Dunbar 1975; Kawai *et al*. 1983
Hamadryas baboon (*Papio hamadryas*)	Kummer 1984; Swedell 2006
Guinea baboon (*Papio papio*)	Galat-Luong *et al*. 2006; Sharman 1981
Modern human (*Homo sapiens*)	Hamilton *et al*. 2007; Layton *et al*. 2012
*Primate species for which multilevel organization has been assumed*	
Grey-shanked douc (*Pygathrix cinerea*)	Ha 2011
Capped langur (*Trachypithecus pileatus*)	Stanford 1991
Golden langur (*Trachypithecus geei*)	Mukherjee and Saha 1974
Banded surili (*Presbytis* cf. *melalophos*)	Bennett 1983
Pig-tailed macaque (*Macaca nemestrina*)	Robertson 1986
Mandrill (*Mandrillus shinx*)	Hoshino *et al*. 1984; *cf*. Abernethy *et al*. 2002
Drill (*Mandrillus leucophaeus*)	Gartlan 1970; see also Astaras *et al*. 2008
Red uakari (*Cacajao calvus ucayalii*)	Bowler and Bodmer 2009; Bowler *et al*. 2012
Golden-backed uakari (*Cacajao melanocephalus*)	Barnett 2005

## Multilevel vs. Fission–Fusion Systems

Multilevel systems are often equated with fission–fusion systems in the literature, specifically with multimale–multifemale (mm–mf) societies with fission–fusion tendencies, as seen in chimpanzees (*Pan troglodytes*). However, we would argue that multilevel systems and fission–fusion are different phenomena. In multilevel societies (which on higher levels also consist of multiple males and multiple females), subunits have stable membership over longer periods and bands are also quite stable in composition. In mm–mf societies with fission–fusion, however, only the higher social grouping level is stable whereas subunits are flexible and unpredictable in terms of size and composition (Chapman *et al*. 1993; Symington 1990). Although multilevel and mm–mf societies are clearly two separate types of primate social organization, fission–fusion does not refer to a social organization *per se* (*pace* Kummer 1971), but describes fluctuating patterns in group cohesion in social mammals, as found for instance in spider monkey (*Ateles* spp.: Symington 1990), chimpanzees (Goodall 1968), long-tailed macaques (*Macaca fascicularis*: van Schaik and van Noordwijk 1988), spotted hyenas (*Crocuta crocuta*: Smith *et al*. 2008), and African buffaloes (*Syncerus caffer*: Cross *et al*. 2005). Orangutans (*Pongo* spp.) have also been included in the category of species with fission–fusion (van Schaik 1999), but they do not form any clearly discernible society. Fission–fusion provides a way of flexibly dealing with costs and benefits of grouping that are largely determined by ecological and sociosexual factors (Chapman 1990; Matsumoto-Oda *et al*. 1998; Sueur *et al*., 2011; van Schaik 1999). Thus, it can be expressed in both mm–mf and multilevel systems, and is better referred to as fission–fusion *dynamics* (Aureli *et al*. 2008; Sueur *et al*. 2011). In multilevel systems, if fissioning occurs, it is along unit lines and leaves the modules intact (molecular pattern) whereas fissioning in other primate social organizations happens in a more random and unstructured way (atomistic pattern). Fission–fusion prevails in most or all multilevel societies, but it is likely more pronounced in hamadryas baboons than in other taxa (Schreier and Swedell in press). It also occurs to varying degrees in Chinese rhinopiths (Table II).

**Table II:** Examples of primate taxa living in multilevel and multimale–multifemale systems with differing degrees of fission–fusion

		Multilevel social organization (molecular)		Multimale–multifemale social organization (atomistic)
		*Strict modularity (stable band composition)*	*Flexible modularity (more fluid bands)*	
Fission–fusion	*Stronger*	*Papio hamadryas*	*Nasalis larvatus*, *Homo sapiens* ^a^	*Brachyteles* spp., *Ateles* spp., *Chiropotes* spp. *Lagothrix* spp., *Pan troglodytes*, *P*. *paniscus*
	*Weaker*	*Rhinopithecus bieti*, *R*. *roxellana*	*Theropithecus gelada*, *Papio papio*	*Macaca fascicularis*, *Piliocolobus* spp.

In multilevel systems, fission–fusion takes place along unit boundaries, with the stable core modules (OMUs) remaining intact (molecular pattern). In multimale–multifemale groups (“classical fission–fusion systems”), fission–fusion occurs in a more random fashion, with regularly changing party composition (atomistic pattern).

^a^The social system of *Homo sapiens* also contains atomistic elements in that foraging parties have changing compositions.

Within multilevel societies, the stability of band membership varies among species. In *strict* multilevel organizations, there are typically two stable and rather closed modules, i.e., the subgroup (OMU or breeding unit) and the larger social group (band), e.g., *Papio hamadryas*. On the other hand, when OMUs congregate on an irregular basis and bands are more fluid and not as consistently assembled or behaviorally integrated as in strict modular systems, this would constitute a flexible system, e.g., *Theropithecus gelada* (Table II; see also Grueter and Zinner 2004).

## Multilevel Systems in Papionins

The hamadryas society is characterized by a hierarchical construction of social units: OMUs, clans, bands, and troops (Abegglen 1984; Kummer 1968, 1984; Schreier and Swedell 2009; Stolba 1979; Swedell 2006). OMUs consist of one adult male and one to several females and their dependent offspring. Clans comprise several (male)-bonded and probably male-related OMUs and also contain solitary males that are unaffiliated with OMUs. Bands most closely resemble the mm–mf group in other primates and comprise several OMUs, including follower and solitary males. Followers are males, often subadults or young adults, that are affiliated with a particular OMU. There is possibly an additional level between the OMU and the clan, at least at one field site in Ethiopia (Filoha; Schreier and Swedell 2012). Bachelor males not attached to any OMU are also members of a clan and band without forming AMUs (Kummer 1968; Pines *et al*. 2011). Troops are temporary nonindividualized aggregations, e.g., when several bands gather at the same sleeping cliff or at scarce waterholes. Most males stay in their natal band and even in their natal clan for life, and male-enforced transfers of females between OMUs occur primarily within the band and more often within a clan than across clans, resulting in OMUs consisting mostly of unrelated females (Sigg *et al*. 1982; Swedell *et al*. 2011). Female–female relationships are poorly developed when compared to those in savanna baboons (but see Swedell 2002). The female’s attention is focused on the leader male, which uses herding as a means of keeping his females’ allegiance. Males also use herding and physical aggression to transfer females among OMUs (Swedell and Schreier 2009).

There are few interunit interactions between adults in *Papio hamadryas*, but sporadic exchanges of threats between adult animals of different units occur (Kummer 1968). Unit males fight any other male attempting to approach and interact with their females except follower males (Kummer 1990; Pines *et al*. 2011; Swedell and Tesfaye 2003). Grooming between members of different OMUs occurs infrequently and is limited to members of the same clan (Schreier and Swedell 2009). In particular, the solitary males of the same clan will groom each other quite frequently, and clan males can also function as allies in a competitive interaction (Abegglen 1984). Interactions between adult females of different OMUs are mostly prevented by male herding behavior (Kummer 1990).

Savanna baboons (yellow, *Papio cynocephalus*; olive, *P*. *anubis*; and chacma baboons, *P*. *ursinus*) normally live in large and relatively cohesive mm–mf groups that are female bonded: males usually disperse out of their natal group while females remain philopatric. Females are arranged into stable linear dominance hierarchies (Hausfater *et al*. 1982; Silk *et al*. 1999), and form strong social bonds, e.g., grooming relationships, with one another (Silk *et al*. 2006). Males compete for dominance and sexual contact with females (Packer 1979), and males and females form sexual consortships during females’ receptive periods (Smuts 1985). OMUs of olive and chacma baboons have been observed too, but they have been attributed to low baboon population densities and small female groups (Kunz and Linsenmair 2008; Swedell 2011; Whiten *et al*. 1987).

Based on mitochondrial information, the phylogenetic distance between hamadryas and olive baboons is smaller than between hamadryas and chacma baboons (Newman *et al*. 2004; Zinner *et al*. 2009), so we principally compare sociological facets of the former two taxa. The key characteristics that set hamadryas apart from savanna baboons are cross-sex bonding in hamadryas vs. female bonding in savanna baboons, male philopatry in hamadryas vs. male dispersal in savanna baboons, and permanent male herding of a cluster of females in hamadryas vs. transient consortships in savanna baboons (Barton 2000; Henzi and Barrett 2005; Swedell and Saunders 2006). It has been speculated that the strong interfemale alliances (as manifest in grooming networks) in savanna baboons hinder the ability of males to segregate groups into separate OMUs, while male control of females limits the expression of female bonds in hamadryas (Barton *et al*. 1996; Swedell 2002).

Gelada societies are structurally similar to hamadryas societies, with several embedded social levels (Dunbar and Dunbar 1975; Kawai *et al*. 1983), i.e., reproductive units, that are not necessarily equivalent to OMUs, as a considerable number of OMUs include follower males (Dunbar 1984; Snyder-Mackler *et al*. 2012), teams, bands (which are much more varied in their composition than those of hamadryas; see Snyder-Mackler *et al*. 2012), and communities. Based on limited vocal recognition among males in gelada multilevel societies, it has recently been suggested that the OMU represents the gelada group and that the band may not be a true social entity, but rather a simple aggregation of individuals induced by predator threat and/or limited sleeping sites (Bergman 2010). Gelada societies differ with regard to the strength of intra- and intersexual social ties and dispersal regime: reproductive units are made up of a number of females that form long-term alliances (Dunbar and Dunbar 1975). They exhibit linear and stable dominance hierarchies (Le Roux *et al*. 2011). Gelada females generally remain in their natal units and rarely transfer across OMUs within their natal bands (transfer across bands has never been observed) and thus form matrilines of related females (Dunbar and Dunbar 1975; Le Roux *et al*. 2011; Mori *et al*. 2003). In contrast, males m ay transfer across bands to form or join AMUs, but many return to their natal bands to establish OMUs (Dunbar 1984). OMU leaders seldom interact with each other (Dunbar 1983a). Mature males that do not have their own OMUs form stable AMUs that sometimes travel separately from bands (Dunbar and Dunbar 1975). Although an OMU leader usually ignores other leader males (unless their respective females get involved in an interunit contest), his stance toward males of AMUs takes the form of vigilance and antagonism (Bergman 2010; Dunbar 1986; Mori 1979). OMU leader males have been seen to confront AMUs collectively (Dunbar and Dunbar 1975; Mori 1979). Affinitive interactions do not usually occur among members of different units; the only exceptions are adult females and juveniles of some units (especially team members) that occasionally enter into affinitive interactions (Dunbar and Dunbar 1975; Mori 1979; Snyder-Mackler *et al*. 2012).

## Multilevel Systems in Colobines

Asian colobines (Presbytini) exhibit several distinct types of social organization (Grueter and van Schaik 2010): 1) independent OMUs that occupy and defend delineated and exclusive home ranges, e.g., Hose’s langurs (*Presbytis hosei*: Mitchell 1994) and purple-faced langurs (*Trachypithecus vetulus*: Rudran 1973); social units can contain one or two additional males, e.g., dusky leaf monkey (*Trachypithecus obscurus*: Curtin 1980); 2) *multilevel societies*, according to the aforementioned definition; 3) intermediate systems between autonomous OMUs and modular societies, in which OMUs have various relations with other OMUs, i.e., friendly relations with some OMUs and antagonistic relations with others), and show a tendency toward spatial amalgamation, i.e., home range boundaries become blurred as units share the same space and under some circumstances or in some species organize daily activities in a cohesive manner. Such intermediate modularity has been documented in *Trachypithecus pileatus* (Stanford 1991) and *T*. *geei* (Mukherjee and Saha 1974). One might argue that species with partly developed modularity that show mere simultaneous exploitation of resources such as roosting sites and food patches do not merit to be called modular and that a clear cutoff point in assembling frequency is needed to demarcate modularity. However, more research is needed on the spatial arrangements, social dynamics, and interunit interactions in these species before we can draw firm conclusions. In a previous publication (Grueter and van Schaik 2010), we subsumed these intermediate species under modular. For the time being and for sake of this review (and phylogram), we treat them as nonmodular (until further field data shed more light on this). Finally, a few species/populations of Hanuman langurs (*Semnopithecus* spp.) form large coherent mm–mf/mixed-sex groups (Borries 2000; Newton 1988).

In multilevel colobines, relations among units range from tolerant to aggressive and tend to vary with familiarity and by season (Yeager 1992). Units are often held together by moderately strong female bonds or cross-sex bonds, and males typically emigrate from the group when reaching adolescence, but female dispersal has been documented in several multilevel colobines as well (Grueter *et al*. in press-a; Kirkpatrick 2011). Females occasionally groom females of other units (Zhang *et al*. 2006), and researchers have observed infant handling across units (Zhang *et al*. 2012).

## Evolutionary Pathways Leading to Multilevel Societies in Nonhuman Primates

**Fig. 1:**
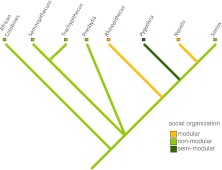
Phylogram showing the occurrence of modular vs. nonmodular social systems in (Asian) colobines. *Pygathrix* is classified as semimodular because the evidence for modularity in this genus is still equivocal (two species have been tentatively described as modular and one as mm–mf (see Table I). Phylograms were constructed in MacClade and phylogenies are based on Perelman *et al*. (2011) and Roos *et al*. (2011).

**Fig. 2:**
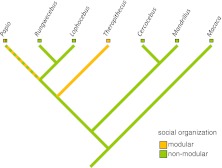
Phylogram showing the distribution of the trait modular vs. nonmodular in papionins. Phylogenies are based on Perelman *et al*. (2011) and Zinner *et al*. (2009).

Two putative historical pathways have been identified for the evolutionary origins of modular societies in primates (Grueter and Zinner 2004). First, the coalescence pathway depicts a scenario whereby OMUs or modules have fused to form the next higher level, e.g., a band. According to phylogenetic reconstructions (Fig. 1), the modular system of some extant Asian colobines —most prominently represented by the snub-nosed monkeys— derives from ancestral species living in single OMUs (Grueter and van Schaik 2010; Grueter and Zinner 2004; *cf*. Yeager and Kirkpatrick 1998). Second, according to the separation pathway, large mm–mf groups have fissioned into modules that are OMUs. This probably applies to certain papionin taxa, such as hamadryas baboons and geladas (Fig. 2). Cladistic comparisons have shown that ancestral gelada and hamadryas baboon forms most likely lived in macaque or savanna baboon-like polygynandrous (“promiscuous”) mm–mf groups that began to dissolve into distinct OMUs with stable mating bonds (Barton 2000; Dunbar 1986; Kummer 1990). In both colobines and papionins, the resulting modular social system thus appears to be a derived feature (autapomorphy). Both suggested evolutionary trajectories are not incompatible with the phylogenetic records of Shultz *et al*. (2011) showing likely transitions from large mm–mf aggregations to single-male harem systems or pair-living and also back transitions from harems to mm–mf groups (and possibly modularity, although the authors did not treat modularity as a distinct class of social organization). The presence of multiple historical pathways leading to modularity in primates may reflect functional heterogeneity, and in the text that follows we will confirm this suggestion (Grueter and Zinner 2004).

## Functions of Multilevel Societies in Nonhuman Primates

Classic socioecological theory considers ecological factors such as food distribution and predation risk as exerting major impacts on the spatiotemporal organization of primate females (and indirectly also of males) and their social relationships, and hence on the social system of a particular taxon (Janson and Goldsmith 1995; van Schaik 1983; Wrangham 1980). The updated socioecological model also includes sexual conflict, in particular coercion of females and infanticide by males, both mediated by life history, as a potentially critical selective factor that shapes grouping and thus the social systems (Chapman and Pavelka 2005; Smuts and Smuts 1993; Sterck *et al*. 1997; van Schaik and Janson 2000). Social organization may also partially be explained by constraining phylogenetic inertia (Di Fiore and Rendall 1994) and thus, low social plasticity, and/or by factors correlated with phylogeny, such as life history. Some mixture of the same factors is likely to be responsible for creating the sort of societal organization we see in the taxa considered here. The question is what selective forces led existing OMUs to form larger bands and what causal factors prompted existing larger mm–mf bands to split into OMUs, respectively?

### A Socioecological Hypothesis for Formation of Multilevel Societies in Colobines

**Fig. 3:**
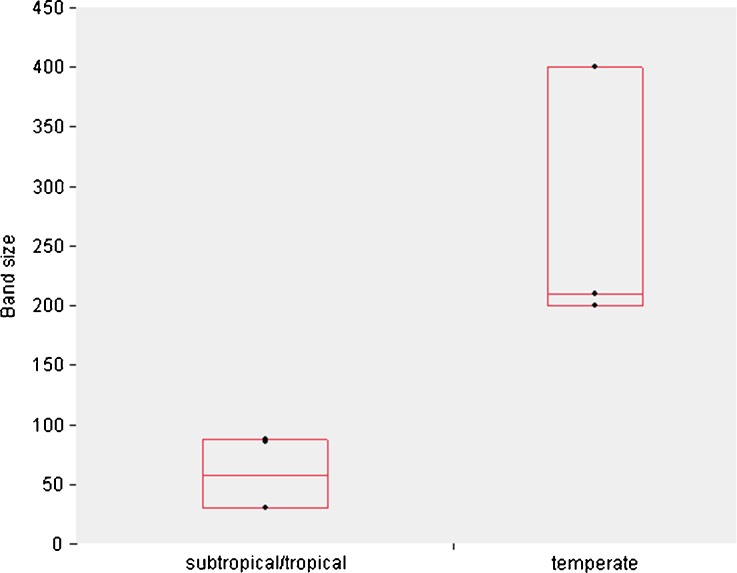
Band sizes in odd-nosed colobines, stratified by habitat. Data are from Grueter *et al*. (in press-a), including the following species: *Rhinopithecus bieti*, *R*. *avunculus*, *R*. *brelichi*, *R*. *roxellana*, *Pygathrix nigripes*, *P*. *cinerea*, and *Nasalis larvatus*. The points represent species means. *n*_temperate_ = 15 groups, *n*_(sub)tropical_ = 7 groups.

For higher-level social associations such as module-based bands to develop, ecological conditions should not have a limiting effect on grouping (within certain margins). First, resources must permit the formation of very large groups, i.e., an abundant and uniformly distributed resource base is required for the formation of bands (Fimbel *et al*. 2001; Rodman 1988). The staple foods of many multilevel colobines appear to be fairly abundant, e.g., lichens in *Rhinopithecus bieti* (Grueter *et al*. 2009; Kirkpatrick *et al*. 1998) or nonephemeral young leaves in *Nasalis larvatus* (Boonratana 1993; Matsuda *et al*. 2009), so the foraging or ranging costs imposed by assembling in bands are most likely relatively insignificant (Grueter and van Schaik 2010). Band size is controlled and constrained by the availability of resources and patch sizes; bands are larger in more temperate forests where foods seem to occur in larger patches (Mann–Whitney *U*, *z* = −3.01, *P* = 0.0027; Fig. 3; see also Kirkpatrick 1998; Kirkpatrick and Grueter 2010). The degree of terrestriality does not seem to have an effect on actual band size (Grueter *unpubl*. *data*). It could be argued that ecological conditions are not merely permissive, but actually drive the nested nature of the multilevel social system of colobines (Matsuda *et al*. 2010), but a detailed review (Grueter and van Schaik 2010) found no evidence for this. The hypothesis that predation has fuelled the formation of superbands is unlikely to apply; as group size benefit from predation quickly saturates (Hamilton 1971), we woud not expect groups of several hundred members, as commonly seen in snub-nosed monkeys (Grueter and van Schaik 2010).

We consider the threat of conspecific males (bachelors) that form AMUs of substantial size and are frequently on the outskirts of the group in most multilevel settings (Grueter *et al*. in press-b; Yeager 1990) to be the most plausible shaping force of multilevel colobine societies. Specifically, we suggest that OMU holders seek one another’s proximity and thereby incur a lower probability of being challenged by bachelor males, either through joint defense or the safety in numbers effect (Bleisch and Xie 1998; Grueter and van Schaik 2010; Rubenstein and Hack 2004). Females would also indirectly benefit from a reduced takeover probability, as this would reduce the risk of infanticide. Increasing the number of coresiding OMUs appears to be functionally comparable to adding more males to a single group. Indeed, primate groups with more males have been shown to be less vulnerable to incursions by nonresident males (Crockett and Janson 2000; Janson and van Schaik 2000; Newton 1986; *cf*. Borries and Koenig 2000). Rubenstein and Hack (2004) showed that as the chance of being harassed by bachelor males increases, stallions exhibit a higher propensity to associate with other stallions to thwart these extra group hazards in plains zebras in multilevel societies. On the other hand, Fischhoff *et al*. (2009) have recently shown that stallion–stallion associations do not show long-term cooperative bonds.

That bachelors pose a significant threat to OMU leaders and females in modular colobines is supported by several lines of evidence: 1) AMUs of substantial size are more or less permanently on the outskirts of the reproductive group in most multilevel settings (Grueter *et al*. in press-b; Hoang 2007; Kirkpatrick 1998; Stanford 1991; Yeager 1990); 2) replacements of resident males are often accompanied by infanticide (Agoramoorthy and Hsu 2005; Ren *et al*. 2011); 3) the frequency of male aggression in a wild group of *Rhinopithecus bieti* was elevated when bachelors were present (Grueter *et al*. in press-b). Additional support for the bachelor threat influencing modularity/aggregation patterns comes from a comparative analysis on Asian colobines that showed that where the expected number of nongroup males is high, units have high home range overlap, show a higher association degree, and have a higher tendency to form bands (Grueter and van Schaik 2010). Researchers have only rarely reported observations that can be interpreted as collective or collaborative aggression of unit males against intruders in modular colobines (Grueter and van Schaik 2010), but some circumstantial evidence has been presented (Zhao and Li 2009). Krzton (2011) observed coordinated patrolling behavior among males of different breeding units in wild *Rhinopithecus roxellana*, vaguely reminiscent of chimpanzee boundary patrols (Wilson and Wrangham 2003). In conclusion, males in modular societies may balance the costs and benefits of associating with potential allies and competitors, and the benefit of enhanced safety when associating with conspecifics may outweigh the costs and have prompted the formation of bands. An interesting case is the *Semnopithecus* population at Jodphur/India where there are solitary OMUs as well as AMUs (Koenig and Borries 2001). Even though the OMU males have to defend their females against takeovers from bachelor males, OMUs do not form a higher social level. It could be that the local ecological conditions (semidesert) do not permit modularity in this case.

Once modularity has been established, female transfer among units within the band may create kinship and tolerance/affinity bridges between units, thus favoring the maintenance of a multilevel system in colobines (*sensu* Chapais 2008). However, data concerning genetic relationships among females in modular colobine societies are yet to be collected. Interunit ties may be further strengthened by infant handling involving females of different units (Zhang *et al*. 2012).

### A Socioecological Explanation for Evolution of Multilevel Societies in Papionins

Modularity in papionins seems to have evolved from ancestral mm–mf groups of considerable size (see earlier). We outline two scenarios, the ecological and the social model, of how large groups might have become substructured. The ecological model assumes that splitting was caused by ecology/environment and social factors came subsequently into play. This model has been generally applied to hamadryas baboons. The alternative, the social model, which we favor here, posits that it was mainly social factors that triggered substructuring, but that ecology was a necessary precondition. An alternative to the ecological model is the time constraint model, and an alternative to the social model is the social brain model, both of which provide other possible explanations for substructuring in papionins.

#### The Ecological Model

It is generally thought that a combination of ecological and social factors promoted the emergence of modularity in papionins (Bergman 2006; Dunbar 1988; Henzi and Barrett 2003, 2005; Kummer 1990, 1995; Swedell and Plummer 2012; Swedell and Saunders 2006). After having colonized a marginally productive semidesert habitat (the habitat in which hamadryas baboons are thought to have evolved), ancestral mm–mf groups began to dissolve into smaller, widely separated foraging units in response to sparse dispersed food resources occurring in small patches and necessitating small group sizes (dispersed resource hypothesis or subgroup hypothesis). Single males could then monopolize these small groups of females and benefit from associating with these units on a long-term basis so as to be able to track female reproductive condition reliably and thereby ensure their access to receptive mates. Selection would have favored aggressive male herding and the emergence of exclusive OMUs to exclude reproductive competitors (other males) and prevent females from mating promiscuously (mate guarding hypothesis, *sensu* Anderson 1983). OMU leaders were then guaranteed reproductive success and high paternity certainty owing to vigorous monopolization. Females also profited from this social, spatial, and sexual constellation because they and their infants received protection from the male against dangers such as infanticide (bodyguard hypothesis, *sensu* Smuts 1985; Wrangham 1987a). Ironically, exclusive monopolization of subgroups of females would have led to an increased risk of infanticide by extragroup males, thereby strengthening the need for male defense of females (Grueter and Zinner 2004; Henzi and Barrett 2003; Swedell and Plummer 2012; Swedell and Saunders 2006). At the same time there was a need for the small units to congregate regularly at certain scarce resources, such as safe sleeping sites and water places (localized resource hypothesis). The distribution of localized resources explains the conglomeration aspect of the hamadryas system.

A similar social feature to the male–female bonds in hamadryas also exists in savanna baboons, i.e., long-term special relationships (“friendships”) between males and females in which the female benefits from male protection (especially against coercion, including infanticide, by other males, and sometimes also against the effects of contest competition among females) and investment in her infant, while the male may benefit from increased mating opportunities with the female, but the evidence for the latter is inconclusive (Nguyen *et al*. 2009; Palombit *et al*. 1997; Smuts 1985). Recent tests, however, using data from hybrid baboons, failed to support the hypothesis that male–female friendships were the precursors to hamadryas male–female bonds. Instead, support was found for the idea that the hamadryas male reproductive strategy represents a “permanent consortship” (Bergman 2006).

Although the ecological model has some explanatory power, several problems remain. The model does not explain the gelada system, unless we assume that ancestral geladas were forced to split into independent OMUs by food constraints, for which there is no supportive evidence. Moreover, several baboon populations in southern Africa live in similarly marginal desert habitats to hamadryas baboons but do not show a hamadryas-like modular social organization (Cowlishaw 1999; Jolly 2007). It is therefore obvious that the ecological model alone is not sufficient to explain the evolution of modularity in baboons.

#### The Time Constraint Model

According to the time constraint hypothesis (Dunbar 1992b), the maximum tolerable group size of savanna baboons in different habitats depends on environmental factors such as the rainfall pattern. If group size becomes too large for a particular habitat, groups exhibit signs of ecological stress, i.e., individuals have to spend more time foraging and less time resting and in social activity. Intragroup competition and the concomitant need for coalitionary support among females increases, while the time a female can allocate to service the relationships on which this support is based is reduced. When a female cohort grows too large, it is no longer possible for females to sustain alliances, causing substructuring of the group and finally group fission. Could a similar reasoning account for the formation of a multilevel social system in geladas and hamadryas baboons? Whatever the ecological reason for the formation of large aggregations in these two species, e.g., predation risk, safe sleeping sites, such large groups can be assumed to be beyond the limits within which females can manage their social relationships in an adaptive manner. However, geladas and hamadryas baboons seem to need to form large groups on a daily or permanent basis, and these large groups are not a result of an increase in population size, as in savanna baboons. Thus permanent fission does not seem an option for hamadryas baboons and geladas. Alternatively, “internal” fission may solve the problems of females by forming small subunits. In such small units females can maintain strong social relationships to ensure coalitionary support in case it is needed.

An extended time constraint model would not explain the internal fission of the hamadryas system. In hamadryas baboons, contest competition for food seems to play a minor role and hence there is no need for coalitionary support among females and female–female bonds are weak (Kummer 1968; Sigg 1980; Swedell 2002). The time constraint model also does not seem to fit geladas very well. Gelada females maintain strong social bonds within small units and exhibit coalitionary support (Dunbar 1980, 1983b), and it has been argued that they compete to gain access to small feeding sites that cannot accommodate many individuals, and that female–female competition intensifies when gelada OMUs coalesce into larger aggregations on open grasslands (Barton 2000). However, given that their primary resource is abundantly available in their habitat (Iwamoto 1979; Nguyen and Fashing, unpubl), female coalitionary bonds may instead be aimed at raising their rank in the hierarchy (Dunbar 1980; *cf*. Le Roux *et al*. 2011, who found that female geladas inherit dominance ranks rather than compete for them).

#### The Social Model

**Fig. 4:**
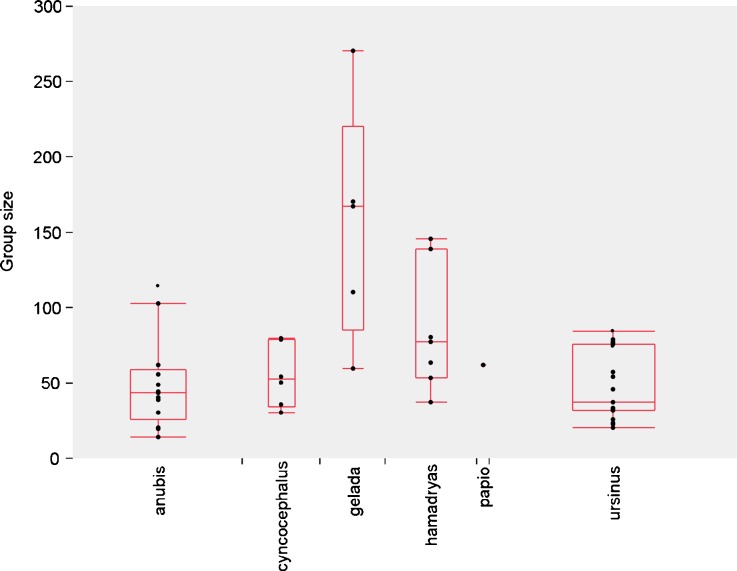
Group/band sizes in different papionin species. The taxa *Theropithecus gelada*, *Papio hamadryas*, and most likely also *P*. *papio* have a multilevel system while the others live in mm–mf groups. The points indicate different groups. Data are from Swedell (2011). *n*_modular_ = 13 groups, *n*_nonmodular_ = 38 groups.

The following hypothetical scenario can be envisaged for hamadryas baboons (Grueter and Zinner 2004; Zinner *et al*. 2001). Large and relatively cohesive social groupings appear for ecological reasons, e.g., reduction of predation risk at scarce sleeping sites and possibly rare waterholes acting as a magnet for units (localized resources and predation avoidance hypotheses). Another scenario for how hamadryas groups could have become so large is given by Jolly’s (2009) frontier hypothesis: during northward extension of range of ancestral baboons, neighboring groups were few and distant in those frontier areas, thus favoring male philopatry. The resulting risk of not finding suitable, unrelated males in the natal group may then have promoted the development of very large social groups. In any case, these large social assemblages bear a cost in terms of enhanced aggression potential and begin to partition for social reasons. The danger of being harassed or attacked by an unfamiliar individual might be greater than in a normal sized mm–mf group of savanna baboons. In particular, the presence of a large number of unfamiliar males may pose a threat of sexual coercion (especially when females are in estrus) and possibly infanticide for females. In large groups with a large number of unfamiliar individuals, social stress may reduce fecundity in females. In such a situation, other females may not be effective coalition partners and a female–female social network would not represent the best solution to this problem. Female–female relationships may be weakened, and females fare better when they join a guarding male (bodyguard and mate guarding hypothesis), thereby reducing coercion by unfamiliar group members, especially by males. This would trigger a shift from female bonding to cross-sex bonding (*sensu* Byrne *et al*. 1990), which might be an exaggeration of the “friendship” relationships between males and females in other baboon taxa (Palombit 1999, 2009; Smuts 1985). An initially or ancestrally mixed mm–mf group becomes divided into modules (OMUs) with reproductive control for the male. The weakly bonded female network makes it easier for males to establish autonomous units (Barton 2000). Ultimately, it is the demographic change, i.e., an increase in aggregation size due to ecological needs with increasing risk of harassment, which forces females into small stable OMUs. Above a certain size, baboon groups are apt to become substructured. Figure 4 shows that groups (bands) of multilevel papionins (*Papio hamadryas*, *Theropithecus gelada*, most likely also *P*. *papio*) are larger than groups of mm–mf baboons (*P*. *anubis*, *P*. *cynocephalus*, *P*. *ursinus*; Mann–Whitney *U*, *z* = 3.61, *P* = 0.0003).

Dunbar (1986, 1988, 1993) envisaged a similar model for the evolution of present day gelada society out of a *Papio*-like mm–mf group. However, contrary to the hamadryas pattern, in this model it was female bonds that were generated as a means to reduce stress in large gelada groups. Group size increased as an adaptation to open country/plains where these primates faced higher predation risk. Large social groupings or aggregations on a permanent or regular basis (grasslands), which are determined by various ecological needs, such as predation avoidance or optimal habitat use and foraging (localized resources and predation), may result in an insecure social environment. It is then conceivable that large groups split into OMUs because females started to form clusters with closely related females within a larger group, and later a male attached himself to them. Dunbar argues that increased aggression and reduced reproductive output associated with growing group size resulted in females tending to bond together into small matrilineal groups for mutual protection (coalitions) to buffer themselves against the stresses and harassment imposed on them by living in large groups. The alternative, that higher levels of sociality in geladas evolved via a merger of OMUs cannot be discounted, but does not seem to fit well with the phylogenetic relationships. Ohsawa (1979) proposed that lack of antagonistic relations of OMUs with distinct “home areas” permitted amalgamation into fluid bands.

Our portrayal of the evolution of the hamadryas society is derived from Dunbar’s gelada scenario with an important difference: Dunbar highlights the gradual intensification of female–female bonding in geladas, whereas we highlight male–female bonding. This difference reflects the different allocation of social effort within these societies. However, what we need to explain is why strong male–female bonds have not become standard in geladas and why gelada females usually do not select a male as a coalition partner in the face of stress and harassment. It may be that in the beginning the small number of hamadryas females clustering around a male were relatives, but that at a later stage in the evolution of the hamadryas system, males integrated unrelated females into these clusters, breaking up the female network within the unit (Swedell and Plummer 2012). It is also worthy of mention that the gelada system is not exclusively founded on female bonds; some females also form close bonds with individual males (Dunbar 1983c). Nevertheless, the disparate expression of social effort in hamadryas baboons and geladas remains a conundrum requiring further intellectual and empirical scrutiny.

#### The Social Brain Model

It has also been proposed that neocortical size may constrain group size in primates (social brain hypothesis) because it determines the ability to process complex information about social relationships (Dunbar 1992a, 1998; *cf*. van Schaik and Deaner 2003). Large groups of several hundred members may therefore only persist on the condition that smaller closed subunits are formed (in the case of hamadryas baboons and geladas OMUs) in which information transfer is still feasible (Fischer and Zinner 2011). Recent experimental evidence in a wild population of geladas supports this hypothesis. Bergman (2010) found that OMU leader males failed to recognize other males around them vocally, even males with which they associated nearly every day.

#### Summary of the Papionin Pattern

In sum, we give preferentiality to the social model for the evolution of multilevel societies in hamadryas baboons and possibly geladas, i.e., the transition from mm–mf to modular was shaped by intersexual factors to a substantial degree, while food dispersion and localized resources provided the ecological background in a process leading to multilevel societies. Ecology provides a logical explanation for the fission–fusion nature of the papionin social system. More predators cause more band cohesion in hamadryas (Kummer *et al*. 1985), while dispersed resources promote temporary disintegration and localized resources cause union of units (Kummer 1968; Schreier and Swedell 2010). However, the observed fission–fusion dynamic of a hamadryas band in Filoha, Ethiopia was not as pronounced as expected given the seasonal changes in resource availability (Schreier and Swedell in press) suggesting that other factors may also play a role. In geladas, a grass carpet on plateau tops in combination with greater exposure to predators causes units to congregate while the restricted availability of grass on the cliff faces causes the higher groupings to drift apart (Dunbar 1993). Moreover, the presence of AMUs and the threats they cause have been shown to bring breeding individuals closer together (Pappano *et al*. 2011), and from this it can be construed that the higher extent of fission–fusion in hamadryas may also be facilitated by the absence of AMUs.

Once a modular structure has evolved, multiple bonds among males seem to be the framework that holds a hamadryas band together (Kummer 1968, 1984). A lack of female transfer within bands and a lack of clan-based male bonds are likely reasons why gelada bands are not maintained as coherently as hamadryas bands. The only closer spatial and social cohesion is found among gelada units that form teams of two units. It has been hypothesized that these “teams” originate from fissioning of a former OMU (Dunbar and Dunbar 1975; Kawai *et al*. 1983; Ohsawa 1979; Snyder-Mackler *et al*. 2012). Related females, although found in two different OMUs after a fission, would hold teams together by associating.

## Multilevel Systems in Hominins

When our definitions are applied to humans, they combine modularity with fission–fusion (Aureli *et al*. 2008). The overwhelming majority of human societies are multilevel (multifamily) groups. A multifamily group is composed of a number of OMUs, monogamous or polygynous (polyandry is rare), interacting with each other on a regular basis. Monogamy is legally enforced in about 17 % of human societies whereas about 80 % exhibit both monogamous and polygynous unions, with the majority of unions being monogamous in any society (Low 2003; Marlowe 2003). Modular communities of monogamous pairs, or mostly monogamous pairs, are a uniquely human phenomenon (Chapais 2011a). Another difference from other modular primate societies is that foraging units in humans are not necessarily OMUs. Human foraging parties are commonly sex-segregated owing to the division of labor, a fundamental and unique feature of human social organization. In other multilevel primate societies foraging units are OMUs and members of one sex do not cooperate in subsistence activities.

Multifamily groups always combine to form more inclusive social entities, which in turn combine to form still more inclusive entities. For example, in hunter-gatherer societies, families form bands ranging in size from 35 to 80 individuals, whose members cooperate in subsistence activities (Gurven 2004; Hamilton *et al*. 2007; Layton and O’Hara 2010; Layton *et al*. 2012; Turnbull 1965), and bands combine to form regional tribes or communities numbering between 250 and 500 individuals who share the same dialect and communal access to fluctuating resources, and gather occasionally for purposes of ritual, politics, trade, exchange of information, gift and mates, sports or warfare (*cf*. Gat 2010; Layton 2008; Layton and O’Hara 2010; Rodseth 2012; Rodseth and Wrangham 2004; Steward 1969). Human societies are thus both federated and nested, and the number of nested levels of organization is in principle unlimited when we consider the whole range of human societies (Chapais 2011b).

Like other primates, humans practice outbreeding through dispersal. However, humans have flexible residence patterns compared to other primates (patrilocality, matrilocality, bilocality, and so on), with many societies exhibiting more than one pattern simultaneously. Thus although dispersal is often sex-biased, it may be bisexual, an uncommon feature in other primates (Chapais 2008). Patrilocality is the majority pattern in human societies as a whole (Murdoch 1981). In hunter-gatherer societies, however, bisexual dispersal is frequent (Alvarez 2004; Hill *et al*. 2011; Marlowe 2004). Male bonding or fraternal interest groups are features of many human societies (Rodseth and Novak 2000; Tiger 1969), but females are also often gregarious and frequently form alliances with other females (Rodseth and Novak 2006; *cf*. Wrangham 1987b), or offer alloparental assistance (Burkart *et al*. 2009; Hrdy 2009).

In contrast with the situation in hamadryas baboons and geladas, bonds between multifamily groups in humans are strong, as are alliances between groups. Basically, this is because individuals maintain bonds with their natal kin even after joining another group to breed contrary to the situation in other primates (Rodseth *et al*. 1991). Correlatively, multifamily groups that are part of the same social entity, e.g., tribe, mingle and interact peacefully. The maintenance of lifetime bonds between dispersed kin creates two categories of social bonds between groups, based, respectively, on consanguineal kinship and affinal (in-law) kinship (Chapais 2008, 2010). First, lifetime bonds between transferred kin and their natal kin create new kinship bonds between the two groups, e.g., between a transferred woman’s children and their maternal uncles and grandparents living in the woman’s natal group. Human kinship networks thus encompass a number of intermarrying groups. Second, the fact that transferred individuals, e.g., women, maintain contact with their natal kin allows their husbands to recognize their wives’ kin. Thus, both spouses are in a position to recognize their in-laws (or affines), which generates affinal kinship bonds. Arguably, the recognition of affinal kinship paved the way for the creation of intergroup mate selection biases observed in human societies, such as levirate (a widow marrying the brother of her deceased husband), sororate (the reciprocal situation), sister exchange, and cross-cousin marriage (Chapais 2008, 2010). This evolutionary conception of the nature of intergroup alliances in humans supports Lévi-Strauss’s (1969) model of reciprocal exogamy (marriage alliance theory), according to which marital unions bind social groups and are a primary form of exchange, although Lévi-Strauss conceived of reciprocal exogamy as a cultural construct devoid of any biological foundation (Chapais 2008, 2010).

Despite the tremendous extent of cultural variation in humans it is thus possible to discern a number of fundamental structural principles that, taken together, embody the uniqueness of human society, its “deep social structure” (Chapais 2011b). This in turn makes it possible to compare human society to the societies of our closest living relatives, chimpanzees, bonobos (*Pan paniscus*), and gorillas (*Gorilla* spp.). We briefly review those social systems to prepare the ground for the phylogenetic reconstruction of human modular societies. Chimpanzees form dynamic mm–mf societies, with communities comprising up to 150 members. Members move around in continually changing parties within the community range (Boesch and Boesch-Achermann 2000; Goodall 1986; Nishida 1990). Females mate with several males, and males with several females (polygynandry) but a common male reproductive strategy is to form a temporary pair-bond or “consortship” with a female for days or weeks, which ensures him near-exclusive sexual access (Tutin 1979). Most females leave their natal communities upon reaching maturity (Nishida *et al*. 2003; Pusey 1979), while males are philopatric and frequently associate and form alliances with one another (Goodall 1986; Watts 2000; Wrangham 1986). Female sociability is generally less pronounced, but varies across populations (Fawcett 2000; Goodall 1986; Lehmann and Boesch 2008; Wrangham *et al*. 1992). Chimpanzees are territorial and intercommunity interactions are usually antagonistic (Goodall 1986; Wrangham 1999), but peaceful intergroup visits of mothers with infants have been recorded (Boesch *et al*. 2008).

Bonobos also form dynamic mm–mf communities comprising up to 75 members (Furuichi 1989) that move around in continually changing parties (Hohmann and Fruth 2002). Males are philopatric and females disperse (Schubert *et al*. 2010), but in contrast to chimpanzees, adult male transfer between communities has been observed (Hohmann 2001). Male–male associations are generally less pronounced when compared to chimpanzees, but vary across populations (Ihobe 1992). The strongest associations are observed between mothers and adult sons and among unrelated females (Furuichi 1997; Hohmann and Fruth 2002; Surbeck *et al*. 2011). Bonobos are less territorial and intergroup encounters are less hostile than in chimpanzees (Kano 1992). Like chimpanzees, they mate polygynandrously.

Gorillas, however, typically live in stable groups of several adult females and one or occasionally more adult males, with a typical size of *ca*. 10 individuals (Robbins 2001, 2011). Females do not usually form strong and persistent bonds with each other, presumably because the abundance of their main food supply obviates the need for alliances among them (Harcourt 1979; Watts 2001). On reaching maturity, females habitually immigrate into new groups or start a new association with a solitary male (Harcourt 1978; Stokes *et al*. 2003). Males also often disperse and become solitary or join bachelor groups (Robbins 1995; Stokes *et al*. 2003), although they may sometimes inherit their father’s group. Groups encounter one another intermittently and typically exhibit displaying behavior when they do so (Robbins and Sawyer 2007). Only very rarely have such encounters led to temporary nonaggressive fusions (in western gorillas) (Bermejo 2004).

## Pathways Leading to the Human Multifamily System

Here, we use principles derived from phylogeny, morphology, paleoecology, and primate socioecology to elucidate the most probable evolutionary sequence leading to the human modular society. We focus on a temporal framework, but acknowledge that allocating a fossil species to a specific social organization is problematic.

### The Phylogenetic Evidence

**Fig. 5:**
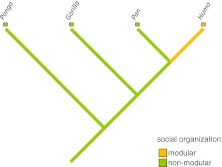
Phylogram depicting the evolution of the trait social organization in hominids. Phylogenies are based on Perelman *et al*. (2011).

Chapais (2008, 2010) discussed likely scenarios for the evolution of the human multifamily system. Based on the evolutionary principle of parsimony, he argued that the ancestral hominin species (the last common ancestor of chimpanzees and humans, LCA) was most likely characterized by a chimpanzee-like mm–mf group composition exhibiting a polygynandrous mating system, female dispersal, male philopatry, and male kinship bonds. Several other essays on early australopithecines/hominins advocate a similar system (Foley and Lee 1996; Ghiglieri 1987; Lovejoy 2009; McHenry 1996; Wrangham 1987b). The multifamily system of humans can then be seen as a derived trait, having emerged through a transition from polgynadry to stable breeding bonds at some point after the *Pan*–*Homo* split (Fig. 5). This scenario is referred to here as the ancestral male kin group hypothesis (Chapais 2008). It is reminiscent of the separation pathway suggested for modularity in baboons.

A second putative evolutionary pathway proposes that the ancestral hominin system was gorilla-like, with a unimale–multifemale group composition, and that the human multifamily system arose from the amalgamation of several such independent polygynous units into a multifamily group, as in our coalescence pathway. A similar socioevolutionary scenario has been put forward by Imanishi (1965) and by Foley and Lee (1996), who considered single gorilla-like OMUs to represent the ancestral state and modular groups with OMUs (hence the multifamily system) the final state, with an additional intermediate state of mm-mf groups (for a slight variant of this see also Geary and Flinn 2001). However, a number of lines of evidence, besides the phylogenetic argument, support the ancestral male kin group hypothesis.

### The Anatomical Evidence

McHenry’s assertion (1992) that early hominins (australopithecines) were anatomically more similar to chimpanzees than to gorillas in terms of absolute body size and relative brain size supports the ancestral male kin group hypothesis (Chapais 2008). The similarity in body mass suggests that early hominins had a diet comparable to that of chimpanzees, which in turn implies that the behavioral ecology, i.e., group size, group composition, ranging patterns, etc. of early hominins may have been chimpanzee-like. While some argue that “the … behavior of early hominins is … unlikely to represent simple amplifications of those shared by with modern apes” (Lovejoy 2009, p. 74), we believe that studies of extant species coupled with strategic modeling, referential models and cladistic analysis can generate crucial information about behavior of extinct hominins (Whiten *et al*. 2009).

Further evidence for the ancestral male kin group hypothesis comes from strontium isotope analysis of tooth enamel in hominins. The strontium contained in plants is incorporated into the teeth of mammals ingesting them during the period of enamel mineralization so that the strontium composition of teeth in mature individuals is a likely marker of the geographical location where those individuals grew up (Copeland *et al*. 2011). The analysis of tooth enamel in 19 South African australopithecines dated around 2 Ma suggests that 50 % of the small individuals (probably females) dispersed from their natal range, whereas 90 % of the large individuals (probably males) did not (Copeland *et al*. 2011). This is compatible with the male philopatry pattern of chimpanzees but not with the dispersal pattern of gorillas where both sexes disperse.

Finally, data on sexual dimorphism in early hominins are compatible with both a mm–mf social system and an OMU-based modular system. It has long been recognized that pronounced body size dimorphism in pre-*Homo* fossil hominins is suggestive of high levels of male–male competition (Leigh 1995; Plavcan and van Schaik 1997; Wolpoff 1976). McHenry (1992, 1996) provides data suggesting that body mass dimorphism in early hominins ranged from comparable to a chimpanzee in *Australopithecus robustus* to quite gorilla-like in *A*. *boisei*. Skeletal dimorphism in *Australopithecus afarensis* is moderate, greater than in *Pan* and similar to that in *Gorilla* (Gordon *et al*. 2008; *cf*. Reno *et al*. 2003). White *et al*. (2009) argued that the primitive *Ardipithecus ramidus* had minimal body size dimorphism, but postcranial fossil evidence is fragmentary. Sexual dimorphism tends to be highest in OMU-based groups, e.g., gorilla, and in modular societies (Grueter and van Schaik 2009) and intermediate in mm–mf groups, e.g., chimpanzee (Plavcan 2001). The relatively high degree of sexual dimorphism in body size in early hominins could be reconciled with either an OMU-based modular social system or a mm–mf system. However, using dimorphism to infer behavior in early hominins is complicated by the unique combination of minimal canine size dimorphism, indicating a lack of male–male competition, and strong body mass dimorphism, consistent with intense male–male competition (Plavcan 2001; Plavcan and van Schaik 1997).

### The Paleoecological Evidence

Several lines of evidence, such as isotopic composition of soil samples and teeth, signify that partly forested environments, particularly woodlands, represented the main habitats for late Miocene hominins (Elton 2008; WoldeGabriel *et al*. 2001), and therefore possibly for the LCA of the chimpanzee/bonobo–human clade, which has been tentatively dated at 6–8 million years ago (Steiper and Young 2006), but for which there is still no clear taxonomic/fossil candidate. Most *Australopithecus* and *Kenyanthropus* (4.2–3.0 mya) and *Paranthropus* (3.0–2.5 Ma) probably lived in rather open mosaic habitats, with open woodlands, bushlands, riverine forests, and seasonal floodplains (Reed 1997; Zazzo *et al*. 2000).

It is generally thought that group size became considerably larger when hominins began occupying more open savannah environments (Foley and Lee 1989; see also Moore 1992), because grouping reduces the probability of predation in open environments (Dunbar 1988). As the habitat of these hominins was becoming increasingly dry and open, valuable food items were dispersed. Underground storage organs such as roots, tubers, bulbs, and corms have been suggested as possible key components of the diet of early hominins, especially robust australopithecines (Laden and Wrangham 2005; Peters and O’Brien 1981). Under such environmental conditions, the spatial cohesiveness of groups would be compromised and the group would become prone to substructuring (Aureli *et al*. 2008; Foley and Gamble 2009) (dispersed resource hypothesis). They would separate during the day to reduce intragroup feeding competition and increase net food intake. This reminds us of the hamadryas strategy (*ecological model*) where ancestral mm–mf troops are hypothesized to have split into smaller foraging units in response to ecological conditions. The smallest units of the foraging parties may have been were lone individuals (as is sometimes the case among human foragers), labile parties or stable subunits, as in hamadryas. It is unlikely that foraging parties were made up of lone individuals because of the considerable predation threat (Hart and Sussman 2009). Stable subunits are better buffered against predators whereas temporary parties are better buffered against food scarcity/patchiness.

A different ecological force, i.e., localized resources or limited refugia in the increasingly patchy savannah habitat, may have promoted return use of certain areas for sleeping, drinking/feeding or safety, e.g., riverine strips, valleys, sleeping cliffs, water holes etc., and led to band formation. In this scenario, all members of the band would be forced to gravitate toward such locations (Aureli *et al*. 2008) and develop some sort of “agreement” to share sites (see Stammbach 1987 and Sueur *et al*. 2011 for hamadryas). These sites would have acted as “information centers” where information about food sources, etc. could be exchanged and cooperative bonds reinforced (Aureli *et al*. 2008). Frequent use of highly localized resources may ultimately have led to the adoption of home-base sites and “central place foraging” in hominins (Layton and Barton 2004; Marlowe 2006; Moore 1996; Rose and Marshall 1996). Once intermale competition became more relaxed, in line with a reduction in canine size dimorphism, and a wider range of habitats were occupied in the evolutionary transition from *Australopithecus* to *Homo*, daily fusing may have become especially beneficial and fully established. The existence of home bases has been inferred from local concentrations of stone tools, raw stone material, and animal bones (Isaac 1978; *cf*. Binford 1980; Potts 1984).

### The Sociosexual Evidence

Ecological parameters probably acted in tandem with sociosexual pressures in the creation of stable families within larger social units, as seen in humans and prehumans. Chapais (2008) argued that the transition from a promiscuous mm–mf society to the humanlike community of mainly monogamous families was not direct, as is often assumed (Fisher 2006; Kaplan *et al*. 2000, 2009; Lovejoy 1981, 2009), but that an intermediate stage was the multiharem type of structure in which all reproductive units are polygynous, as seen in extant hamadryas baboons and geladas. The evolution of human pair-bonding is thus hypothesized to have taken place in two steps. The first step involved a shift from the sexually promiscuous mm–mf group to the multiharem group: from sexual promiscuity to polygyny. One possibility is that this shift occurred for similar reasons as substructuring in baboons, i.e., through the interplay of the tendency of males to monopolize females and the effect of ecological constraints on the spatial distribution of females. Specifically, male monopolization potential is higher when females forage in small (widely spaced) groups, which is itself caused by the density and spatial dispersion of food (dispersed resource hypothesis and mate guarding hypothesis). Stable breeding bonds in hominins likely resulted from tradeoffs between male sexual competition and female feeding constraints, as has been demonstrated for other mammals. An alternative possibility for the transition from sexual promiscuity to a multiharem structure is the bodyguard hypothesis, which posits that females establish a long-lasting sociosexual (pair) bond with a particular male that can act on behalf of the female as a “bodyguard” or “hired gun” to counter coercion (sexual harassment, infanticide) from outside conspecific males (Blurton Jones *et al*. 2000; Mesnick 1997; Palombit 1999; Rodseth and Novak 2006; Smuts 1992; van Schaik and Dunbar 1990). Paradoxically, male mate guarding can sometimes take the form of sexual aggression such as rape (Emery Thompson 2009; Smuts 1992). Wrangham (2009) and Wrangham *et al*. (1999) proposed a credible scenario that sees the male–female pair-bond as a reciprocal arrangements in which males can count on a woman for cooking and providing an evening meal at camp and females rely on male protection from theft of gathered and cooked foods. Females may associate closely not only with a protective male, but also with related females for the purpose of cooperative infant rearing (Hrdy 2009; Swedell and Plummer 2012).

Whatever the exact process involved, several lines of evidence support an intermediate polgynandry-to-polygyny sequence rather than the direct polgynandry-to-monogamy sequence. First, many male primates monopolize more than one female, this resulting in a preponderance of polygynous mating systems and an underrepresentation of monogamous systems (Fuentes 1999). Second, polygyny is the rule in all other multilevel primate societies; modularity based on monogamous pairs has not been documented in nonhuman primates. Presumably this is because the spatial cohesion of females in large mixed-sex groups always makes polygyny feasible (Chapais 2011a). Accordingly, the various models discussed in the preceding text in relation to the evolution of multilevel societies in papionins (ecological model, time constraint model, social model, social brain model) have in common that they predict the fragmentation of large mm–mf groups into polygynous units, not into monogamous ones. Third, the high degree of sexual dimorphism in body size in early hominins argues against monogamy in these species (see earlier). Fourth, polygyny is practiced in the majority of human societies, which suggests that it was an integral part of the human species’ evolutionary past, if not the ancestral hominin mating system (Chapais 2008). Fifth, the view that monogamy evolved directly from polgynandry typically associated with the idea that monogamy evolved in response to an increase in the dependency of children and to selective pressures for the evolution of paternal investment and male provisioning (Fisher 2006; Kaplan *et al*. 2000, 2009; Lovejoy 1981, 2009). Such a view, however, runs counter to our knowledge of the evolution of monogamy and paternal care in mammals in general (Chapais 2008). Specifically, phylogenetic analyses of mammalian mating and parental care systems indicate that paternal investment evolved *after* pair-bonding was already established (Brotherton and Komers 2003; van Schaik and Kappeler 2003). The role of paternal care in the evolution of pair bonds has also been questioned on other grounds (Hawkes 2004).

The second step in the evolution of the human mating system was the transition from the multi-OMU structure to the multimonogamous family structure in which most families are monogamous and some are polygynous. It is noteworthy that this sequence markedly reduces the number of scenarios and explanations concerned with the origin of monogamy because it implies that monogamy evolved through a reduction in the number of females monopolized by the average male, in other words, as a result of the evolution of constraints on polygyny (Chapais 2008). One possibility, labeled the provisioning hypothesis, explains the transition in terms of the benefits of male provisioning for mothers and offspring by positing that it became progressively more advantageous for a mother to be the single beneficiary of a male’s provisioning effort. For males to reduce the size of their family units and forego mating opportunities for paternal investment, we must assume a substantial reduction in levels of sexual competition between males. This hypothesis therefore predicts generalized monogamy (little polygyny) in human societies (Chapais 2011a). Another possibility, the leveling hypothesis, states that after the evolution of lethal weapons —a uniquely human feature that equalized the competitive abilities of males— it became much more costly for males to monopolize more than one female. The consistent exclusion of a large fraction of males from the pool of reproductive individuals, as observed in, e.g., hamadryas baboons, would have become prohibitive and would likely have given way to some sort of polygyny–monogamy mix, with most males being monogamously mated and just a few males forming polygynous units. The importance of polygyny in human societies is more compatible with the leveling hypothesis (Chapais 2011a). The majority of human societies allow polygyny (Murdoch 1981), but its frequency depends on subsistence style: monogamy predominates in forager societies, whereas pastoralists and agriculturalists show significant polygyny (Kaplan *et al*. 2009). So, although polygyny would antedate monogamy evolutionarily, polygyny could reappear secondarily after the adoption of a system based on agriculture and land tenure which generated socioeconomic inequities (Chapais 2011a; Fisher 1989).

Based on the foregoing arguments we propose the following scenario for the evolution of modular societies in hominins. The LCA initially lived in large mm–mf groups with fission–fusion. Substructuring into polygynous units evolved because it minimized feeding competition during the day in a patchy environment, allowed males to adopt a stabilizing reproductive strategy (mate guarding hypothesis), and made it possible for females to retain a protector male (bodyguard hypothesis). Moreover, male monopolization of isolated units was also facilitated in a habitat of dispersed resources (also mate guarding hypothesis). LCA parties/subunits were prone to coalesce around localized drinking/feeding areas and scarce refugia in an open habitat (localized resource hypothesis and predation avoidance hypothesis), factors that promoted band formation. Polygynous subunits subsequently evolved into mostly monogamous units owing to the evolution of constraints on polygyny. Pair-bonding might have later operated as a preadaptation for the evolution of paternal care and the sexual division of labor in response to the evolution of protracted juvenile dependency and high costs of maternity (Chapais 2011a).

## Conclusions

**Fig. 6:**
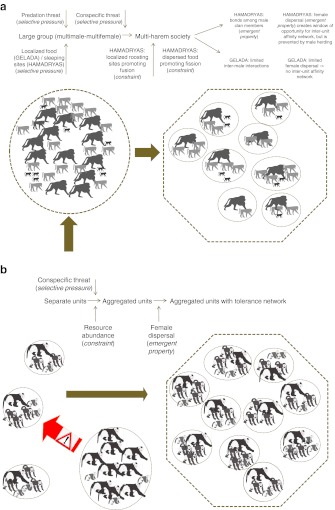

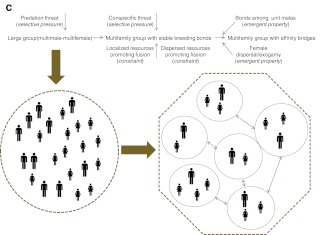
Putative evolutionary pathway leading to multilevel sociality in papionins (**a**), snub-nosed monkeys/odd-nosed monkeys (**b**), and humans (**c**). (Baboon pictograms by P. Henzi, snub-nosed monkey pictograms by K. Meisterhans and C. C. Grueter.)

In this article, we have attempted to typify modular societies as a distinct form of primate sociality. We have also emphasized that fission–fusion and modularity should be treated as distinct phenomena that can be coupled, but do not have to be. We have developed a theoretical framework based on socioecological theory to picture parsimonious scenarios for the evolution and maintenance of these most complex social systems in a variety of primate lineages. One main point is that the precursors to modularity seem to have been large mixed-sex groups in hominins and baboons that subsequently underwent internal fission, but autonomous OMUs that coalesced into bands in colobines (summarized in Fig. 6). We have provided some evidence for the importance of conspecific threat as a potentially shaping selective force in the evolution of multilevel societies in primates. In colobines and geladas, the threat would originate from AMUs at the margin of the group, while in humans and hamadryas it would come primarily from within the bisexual group. This conspecific threat acted in tandem with the ecological setting, resulting in the formation of a modular society.

Two unifying elements favoring the maintenance of modular societies are, depending on the species, male cooperation and female dispersal. That males cooperate to compete against other males is widely accepted as a universal human attribute (Brown 1991), and it is reasonable to assume that male bonds play a part in promoting the maintenance (as opposed to the evolution) of multilevel societies. While the conjugal ties of the pair bond are often seen as a hallmark of human social evolution, a major countervailing element of human social organization are “fraternal interest groups” that are power groups of related males. Rodseth and Novak (2000, 2009) and Rodseth (2012) have made a strong case for the ubiquity of all-male associations in human societies which in extreme cases can shift the conjugal family to a mere peripheral element of the band. Co-residence of several nuclear families in bands and meta-bands facilitates the recruitment of male allies for defense and warfare against enemy groups (Rodseth 2012) as well as cooperative hunting (Hill 2002). The evidence for intermale cooperation in our multilevel cousins is still patchy. We need a better understanding of how relations among unit males are manifested in geladas, hamadryas baboons, and colobines and if they show cooperation (be it based on kin selection, reciprocal altruism, or mutualism) and how important bonds and coalitions are in keeping higher-level groups together and enhancing the fitness of group members. The so-called coalitionary traits metric —which classifies various degrees or intensities of coalitionary behavior as a continuous function based on the presence of mutual tolerance, collaboration, and partner preferences (Olson and Blumstein 2009)— might provide a good starting point to differentiate the coalitionary tendencies of males in multilevel societies. Also poorly investigated is how kinship and inclusive fitness benefits shape the nature of spatial arrangements of males in groups and specifically male–male bonding patterns.

The other unifying element is female dispersal. In colobines, female dispersal within bands likely contributes to interunit tolerance and sporadic affiliation among females and thereby fortifies the maintenance of a modular society. In hamadryas, female dispersal, mainly within band and forcefully executed by males (Swedell *et al*. 2011), is the norm and would create a window of opportunity for interunit affiliation based on affinal kinship and consanguineal kinship between females of the same clan, but the latter is often prevented by male herding (Kummer 1990). In geladas, lack of female dispersal is probably responsible for the lack of an affiliation network, with the exception of teams. The role of female exogamy in supporting the maintenance of a multilevel system by generating kinship and affinity bridges between interbreeding groups (Chapais 2008; Rodseth *et al*. 1991) needs to be more fully explored and integrated into the theoretical framework developed here. Humans are the only multilevel species in which mere tolerance between interbreeding groups evolved into multigroup cooperative networks and the coordination of whole social groups (Chapais 2011b; Rodseth *et al*. 1991), giving rise to many derived features of human sociality such as intense cooperation, prosociality, and cultural transmission (Hill *et al*. 2011).
